# Identification of misdiagnosed HIV clients in an Early Access to ART for All implementation study in Swaziland

**DOI:** 10.7448/IAS.20.7.21756

**Published:** 2017-08-29

**Authors:** Shaukat Khan, Emma Mafara, Munyaradzi Pasipamire, Donna Spiegelman, Sikhathele Mazibuko, Nombuso Ntshalintshali, Anita Hettema, Charlotte Lejeune, Fiona Walsh, Velephi Okello

**Affiliations:** ^a^ Clinton Health Access Initiative (CHAI), Mbabane, Swaziland; ^b^ Department of Epidemiology, Ministry of Health, Mbabane, Swaziland; ^c^ Harvard T.H. Chan School of Public Health, Boston, MA, USA; ^d^ Clinton Health Access Initiative (CHAI), Boston, MA, USA

**Keywords:** Early Access to ART for All, HIV misdiagnosis, HIV false positive, treatment for all, Universal test and treat, Swaziland, HIV testing

## Abstract

**Introduction**: Rapid diagnostic testing has made HIV diagnosis and subsequent treatment more accessible. However, multiple factors, including improper implementation of testing strategies and clerical errors, have been reported to lead to HIV misdiagnosis. The World Health Organization has recommended HIV retesting prior to antiretroviral therapy (ART) initiation which has become pertinent with scaling up of Early Access to ART for All (EAAA). In this analysis, misdiagnosed clients are identified from a subgroup of clients enrolled in EAAA implementation study in Swaziland.

**Methods**: The subgroup to assess misdiagnosis was identified from enrolled EAAA study clients, who had an undetectable viral load prior to ART initiation between September 1, 2014 and May 31, 2016. One hundred and five of 2533 (4%) clients had an undetectable viral load prior to initiation to ART (pre-ART). The HIV status of clients was confirmed using the Determine HIV 1/2 and Uni-Gold HIV 1/2 rapid tests performed serially as recommended by the national testing algorithm. The status of clients on ART was additionally confirmed by fourth-generation HIV Ag/Ab combo tests, Architect and Genscreen Ultra.

**Results**: Fourteen of the 105 (13%) clients were false positive (HIV negative) on confirmation testing, of whom five (36%) were still in pre-ART care, while nine (64%) were in ART care. Overall, proportion of false positive was 0.6% (14/2533). The false-positive clients had a median CD4 of 791 cells/ml (interquartile range (IQR): 628, 967) compared to 549 cells/ml (IQR: 387, 791) for true positives (HIV positive) (*p* = 0.0081) and were nearly 20 years older (*p* = 0.0008).

**Conclusions**: Overall 0.6% of all enrolled EAAA clients were misdiagnosed, and 64% of misdiagnosed clients were initiated on ART. With adoption of EAAA guidelines by national governments, ART initiation regardless of immunological criteria, strengthening of proficiency testing and adoption of retesting prior to ART initiation would allow identification of misdiagnosed clients and further reduce potential of initiating misdiagnosed clients on ART.

## Introduction

**Figure 1.**
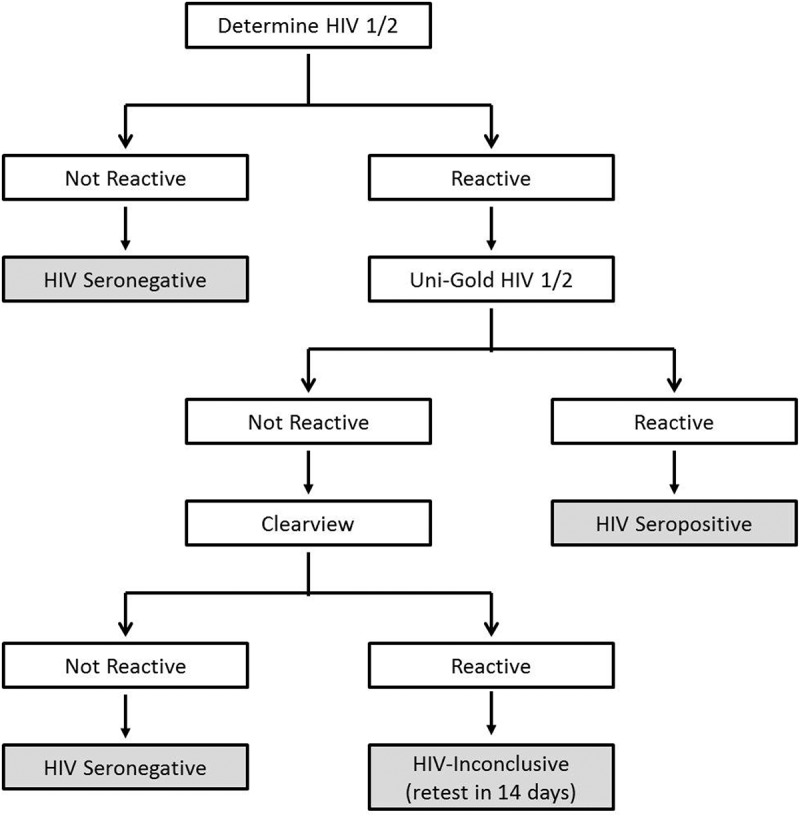
National testing algorithm. The flow chart depicts Swaziland’s national testing algorithm for determination of HIV status. According to the national testing algorithm, a non-reactive first RDT is reported as HIV negative. In case of a reactive first RDT, a second RDT is conducted. If this second RDT is reactive, it is reported as HIV positive. However, if the result is non-reactive, a third RDT, Clearview COMPLETE HIV-1/2 (Chembio Diagnostic systems, Inc., Medford, NY, USA), is conducted. Non-reactive Clearview tests are reported as HIV negative while a reactive Clearview is reported as HIV inconclusive. Clients with inconclusive results are given another appointment to come after two weeks for a retest.

HIV is the leading public health concern in Swaziland with HIV prevalence of 32% and annual incidence of 2.4% among 18–49-year-old adults as determined by the 2011 Swaziland HIV Incidence Measurement Survey (SHIMS) [1]. SHIMS also identified substantial differences in prevalence among women (39%) and men (24%) and additionally reported that 38% of HIV-infected individuals were unaware of their HIV status [1]. The SHIMS data highlighted the need for an improvement of HIV testing and preventive care services in Swaziland. As effective HIV screening is critical for the identification of HIV-positive clients and their subsequent enrolment into antiretroviral therapy (ART) care, Swaziland has adopted the 2012 World Health Organization (WHO)-recommended HIV testing strategy for high-prevalence countries: the use of two rapid diagnostic tests (RDTs) [2]. In Swaziland’s national HIV testing algorithm, Alere Determine HIV-1/2 Ag/Ab Combo (Alere Inc., Yavne, Israel) is used as the first RDT and Uni-Gold HIV test (Trinity Biotech, Bray, Ireland) as the second RDT (Figure 1).

The RDT-based testing algorithm has been an essential tool for the diagnosis of HIV infection. In 2014 alone, the WHO reported that approximately 150 million individuals across 129 low- and middle-income countries have been tested for HIV, in large part due to availability of RDT [3]. The RDT-based testing algorithm has decentralized HIV testing services from laboratory settings to non-laboratory settings such as non-lab service points within facilities, community outreach campaigns and door-to-door testing. Although RDTs have been shown to be highly reliable for HIV diagnosis [4,5], recent reports have highlighted a substantial level of false-positive diagnoses in audits of testing programmes [3,6–8]. In addition, survey and clinical trials have also identified false positives, albeit only in a subset of the retested samples [9–11].

In 2015, the WHO released new adult treatment guidelines that recommended offering ART as soon as possible following diagnosis [12]. As countries adopt these new guidelines, there is renewed scrutiny to ensure the validity of HIV testing programmes. Since 1997, WHO has been recommending retesting prior to ART initiation; however, a 2015 review of 48 national HIV testing policies found that only two programmes included testing before ART initiation in their national guidelines [13]. As of April 2017, Swaziland has not adopted retesting prior to ART initiation.

This analysis reports on the identification of misdiagnosed clients enrolled into an Early Access to ART for All (EAAA) implementation study.

## Methods

### Study population

The Maximizing Antiretroviral Therapy (ART) for better health and zero new infection (MaxART) EAAA implementation study was designed to determine the feasibility, acceptability, clinical outcomes, affordability and scalability of offering early antiretroviral treatment to all HIV-positive adults in Swaziland’s public sector health system. The trial uses a randomized stepped-wedge design across seven paired facilities (13 primary healthcare clinics and 1 regional hospital) in Hhohho Region. The trial includes all HIV-positive and self-reported ART-naive adults ≥18 years of age, not pregnant or breastfeeding and able to give oral consent for an additional blood draw at enrolment and ART monitoring.

### Study sample

**Figure 2.**
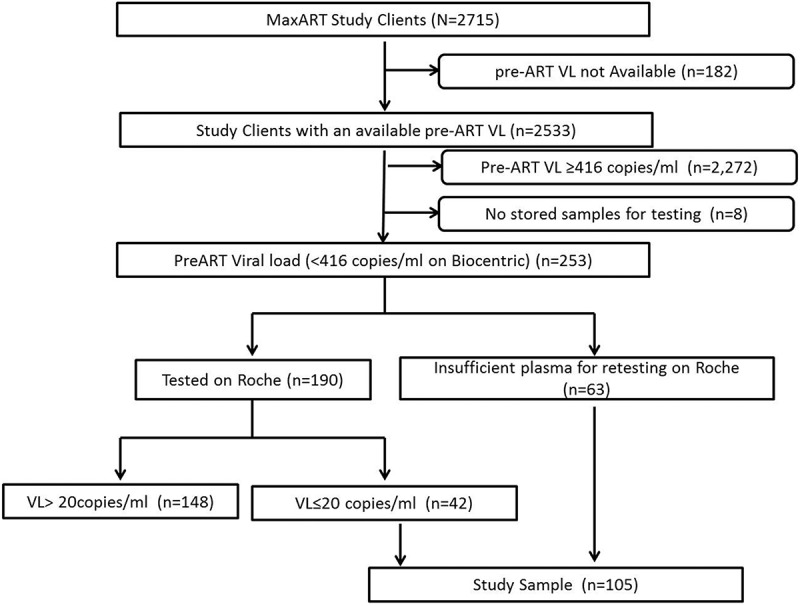
Procedure used to identify study sample for HIV status confirmation. This flow chart depicts the procedures used to identify the sample population from the study population. The clients identified for HIV status confirmation testing were found to have an undetectable viral load on the Biocentric platform and/or on Roche platform at pre-ART enrolment.

This paper describes the analysis of samples collected at the 14 study participation sites between September 1, 2014 and May 31, 2016. During this period, 2715 clients were enrolled in the study, of which 2533 (93%) had a pre-ART viral load (Figure 2). One sample of 6 ml of ethylenediaminetetraacetic acid (EDTA) anticoagulated blood was collected from each consenting client, which was used to prepare a plasma sample that was tested for viral load on the Biocentric platform (Bandol, France) (Instruments: Nordiag Arrow and Bio-Rad CFX 96 real-time detection Assay: Generic HIV Charge Virale). Any remaining plasma from this sample was then frozen at −80°C and stored at the National Reference Laboratory (NRL), Mbabane, Swaziland. Of 2533 clients, 253 (10%) of the clients had an undetectable pre-ART viral load and a stored frozen plasma sample (Figure 2). To exclude failure of viral RNA detectability due to the higher threshold of the Biocentric Platform (<416 copies/ml), samples were retested on the Roche Molecular Diagnostic viral load platform (Pleasanton, California, USA) (Instruments: COBAS AmpliPrep/COBAS Taqman System (CAP/CTM); Assay: CAP/CTM HIV-1 Test V2.0), which has a 20 copies/ml minimum detection threshold. As the Roche viral load protocol requires minimum of 1 ml plasma for testing, 190 of 253 (75%) samples were tested on the Roche platform (Figure 2) of which 42 of 190 (22%) samples were undetectable on Roche and were included for confirmation of HIV Status. Remaining 63 of 253 (25%), which did not have sufficient sample for retesting on the Roche platform, were also included for confirmation of HIV status (Figure 2). A total of 105 of 2533 samples (4%) were identified for confirmation of HIV status (Figure 2).

### National testing algorithm on frozen plasma samples

The clients’ frozen plasma samples from the initial pre-ART viral load were retested at the NRL, using the Swaziland national testing algorithm, to determine HIV status (Figure 1).

### Confirmation of HIV status

Clients testing non-reactive or indeterminate were requested for an additional blood draw. Two separate procedures were followed based on whether the client had been initiated on ART or not at the time of redraw.

For pre-ART clients, a fresh blood sample was drawn, and clients’ HIV status was confirmed using the Swaziland national testing algorithm at the National Referral Laboratory (Figure 1). Clients who were dual reactive on Determine and Uni-Gold were interpreted as true positive (HIV positive) and non-reactive on Determine as false positive (HIV negative).

For ART clients, similar to pre-ART clients, a fresh blood sample was drawn, and clients’ HIV status was determined using the Swaziland national testing algorithm. As clients were on ART, additional testing was conducted using fourth-generation antigen/antibody (Ag/Ab) tests for HIV status confirmation. Fourth-generation tests, Abbott Architect HIV Ag/Ab combo assay (Abbott, Wiesbaden, Germany) and BioRad Genscreen Ultra HIV Ag/Ab combo assay (Bio-Rad, Marnes-la-Coquette, France) were conducted at the National Institute of Communicable Diseases in Johannesburg, South Africa. Clients who were dual reactive on the fourth-generation test were interpreted as true positive (HIV positive) and dual non-reactive as false positive (HIV negative).

### Clinical characteristics of the clients

Clients’ demographic and CD4 cell count results were obtained from the study database, which was extracted from the Ministry of Health’s standard chronic care patient files as part of the EAAA study procedures.

### Analysis

Statistical analysis was conducted using Stata version 12 (StataCorp, College Station, TX, USA).

### Ethical considerations

The MaxART EAAA implementation study was approved by the Swaziland National Health Research Review Board in July 2014 (Reference Number: MH/599C/FWA 000 15267) and is registered on ClinicalTrials.gov with ID NCT02909218. Verbal consent was obtained from all study clients for collection and testing of blood samples in accordance with the approved protocol.

## Results and discussion

Pre-ART viral load results were available for 2533 clients. Among those, 105 (4%) were below the limit of viral load detection on the Biocentric and/or Roche viral load platform. Previous studies have reported less than 1% of untreated HIV-positive individuals with an undetectable viral load [14,15]. However, the viral load platforms have become increasingly sensitive which could explain discrepancy in proportion of clients reported to be undetectable in previous studies and in current study. Even within this study, a difference in detectability due to threshold levels using Roche platform with 20 copies/ml versus Biocentric 416 copies/ml is evident.

### Identifying potential misdiagnosed clients

**Figure 3.**
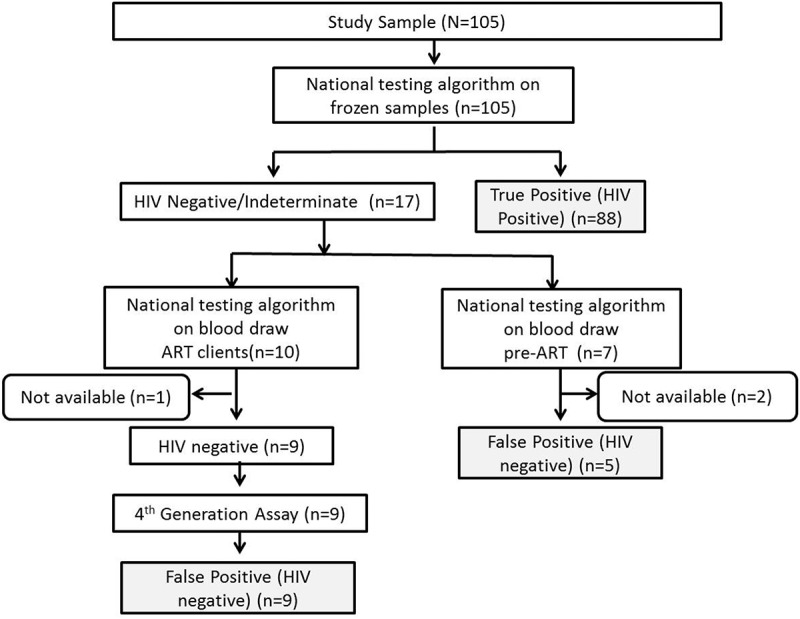
Testing procedure used to classify viral load undetectable clients as true positive (HIV positive) and false positive (HIV negative). Clients were classified as currently on ART or pre-ART at the time of their blood redraws. All clients with stored frozen samples prior to ART initiation were assayed using national testing algorithm. Clients who were determined to be HIV negative and/or indeterminate were requested for an additional blood draw to confirm their HIV status and the national testing algorithm was repeated on the blood redraws. If the client was on ART at the time of blood draw, the samples were further tested in parallel on Architect HIV Ag/Ab and Genscreen Ultra HIV Ag/Ab.

Retesting of clients with an undetectable viral load resulted in confirmation of 88 of 105 (84%) clients as HIV positive. Sixteen of 105 (15%) clients were determined to be HIV negative and 1 (1%) client inconclusive, resulting in identification of 17 of 105 (16%) client for further HIV testing (Figure 3).

### Confirmation of misdiagnosis

As initial testing was done on a frozen sample, a blood draw was requested from the 17 clients identified for further testing (Figure 3). At the time of the request for blood draw, 7 of 17 (41%) clients were still in pre-ART care, while 10 of 17 (59%) had initiated ART.

Samples were redrawn from five of seven (71%) pre-ART clients, and all of them were false positive, according to the Swaziland national testing algorithm. Two (29%) pre-ART clients were lost to follow-up and were unable to be contacted for HIV status confirmation.

Samples from 9 of 10 (90%) ART clients were redrawn, and all of them were determined to be false positive, as they were HIV negative on repeat of the national testing algorithm on their pre-ART sample as well as on additional fourth-generation Ag/Ab. One (10%) ART client was unable to be contacted for redraw for confirmation testing as they were lost to follow-up.

Overall, 14 of 105 (13%) clients were identified as false positive. The false-positive (*n* = 14) clients represent 0.6% of the subset of all clients (*n* = 2533), which is considerably lower in comparison to 10% false-positive rate in Democratic Republic of Congo (DRC), 5% in Ethiopia and 3% in Burundi as determined from evaluation studies [6,7]. In Malawi, programmatic data have shown that 2% (547/30,300) of confirmatory tests could not be conclusively diagnosed [16].

### Interviews with misdiagnosed ART clients

Follow-up interviews were conducted with the nine HIV-negative clients on ART to understand their HIV testing experience that led to them enrolling on ART. Six clients said they had tested HIV negative at the time of enrolment into care. Two clients were told that they were HIV positive yet did not see or have their results explained. One tested with their partner and they were shown one HIV-positive test result. These interviews highlighted several potential cofactors leading to their enrolment into care including administrative error, user error and clients’ circumstantial belief that they are HIV positive.

### Clinical characteristics of clients

The false-positive clients originated from 9 of 14 (64%) facilities (Table 1) with median age of 52 years old (interquartile range (IQR): 40, 63) compared to 35 years old (IQR: 28, 44) for true positives (*p* = 0.0008) and median CD4 at enrolment of 791 cells/ml (IQR: 628, 967) and 549 cells/ml (IQR: 387, 791) (*p* = 0.0081), respectively (Table 1). Mean difference in days between initial HIV-positive test and study enrolment for clients identified as false positive was 392 days (IQR: 45, 1587) compared to 201 days (IQR: 6, 981) for the true positives (*p* = 0.2132) (Table 1). Twenty-nine per cent of the false positive and 22% of true positive were male (*p* = 0.584) with 14% of the false positive single, 71% were married, while 41% of true positive were single and 53% were married at HIV diagnosis (Table 1).

**Table 1.** Demographic and clinical characteristics of false and true positives

Variable		False positive (*n* = 14)	True positive (*n* = 91)	*p*-Value^c^
Age	Median (Q1, Q3)	52 (40, 63)	35 (28, 44)	0.0008**^d^**
Sex	Male (%)	4 (29)	20 (22)	0.584**^e^**
			
CD4^a^	Median (Q1, Q3)	791 (628, 967)	549 (387, 791)	0.0081**^d^**
Days from date of first reported HIVtest and study enrolment	Median (Q1, Q3)	392 (45, 1587)	201 (6, 981)	0.2017**^d^**
Marital status^b^	Single (%)	2 (14)	36 (41)	0.160**^e^**
	Married (%)	10 (71)	46 (53)	
	Widowed (%)	2 (14)	4 (5)	
	Divorce (%)	0 (0)	1 (1)	
**Facility (%)**				
Facility-1		1 (7)	4 (4)	0.217**^e^**
Facility-2		1 (7)	4 (4)	
Facility-3		3 (21)	8 (9)	
Facility-4		2 (14)	6 (7)	
Facility-5		0 (0)	4 (4)	
Facility-6		2 (14)	2 (2)	
Facility-7		1 (7)	10 (11)	
Facility-8		1 (7)	1 (1)	
Facility-9		1 (7)	8 (9)	
Facility-10		0 (0)	3 (3)	
Facility-11		2 (14)	14 (15)	
Facility-12		0 (0)	13 (14)	
Facility-13		0 (0)	12 (13)	
Facility-14		0 (0)	4 (4)	

^a^Ten clients did not have an enrolment CD4 available.

^b^Four clients did not have marital status recorded.

^c^Missing values omitted in statistical analysis.

^d^Mann–Whitney test.

^e^ *χ* ^2^-test.

This study found that clients with a false-positive result had significantly higher CD4 at enrolment than the true positives, 791 and 541 cells/ml, respectively (*p* = 0.0081), and there was a significant difference in median age among false positive and true positives, with the false positives being nearly 20 years older (*p* = 0.0008). The evaluation programmes in DRC reported a median age of 42 years old with median CD4 of 1107 cells/ml for false positives; however, there was no comparison statistics for true positive reported [7].

### Limitations

The study was not designed to assess the validity of viral load as a diagnostic tool but as a selection tool to identify enrolled clients for investigation for HIV retesting. Our findings show a low proportion of false positives in a subset of clients enrolled in an implementation study over a period of 21 months. As the median time between initial test and enrolment of the study was 392 days (IQR: 45, 1587) and there is a lack of data from the time of the initial HIV test, we are unable to determine the cause of the misdiagnosis. However, systematic reviews have identified several potential causes of misdiagnosis [3,17]. In a WHO report on misdiagnosis, user error, suboptimal testing strategy, cross-reactivity and poor management, and supervision practices were suggested as factors related to less than optimal test specificity [3]. Additionally, programmatic data across different geographical location and time have shown variability in specificity of different RDTs, which can have an impact on false-positive rate [18]. In addition to specificity, low sensitivity of the RDTs has also been reported in field settings due to poor adherence to the recommended testing protocols [19].

Additional limitations of the study include the inability to identify the initial cause of undetectable pre-ART viral load in true positives. Due to lack of sufficient plasma, errors due to sample handling, plasma preparation, lab equipment error or user and administrative errors could not be ruled out. As the clients were self-reported ART naive, it was not possible to verify that they were virally suppressed in the absence of ART.

Enrolment of false positives in HIV care results in needless exposure to long-term ART that is detrimental to an individual’s health and well-being and has potential adverse effects on relationships within their family and social circles. In addition, misdiagnosis creates undesirable wastage and unnecessary burden to the resources at the programmatic level, including health worker time and medical costs. Therefore, assuring and maintaining the quality of HIV testing services and consequently correct HIV diagnosis is an urgent priority, as even more intensive programmes are rolled out and more people are being offered immediate access to treatment without clinical or immunological threshold. Further studies are required to investigate quality of testing and accuracy of HIV testing in context of Swaziland to identify potential causes of misdiagnosis, such as user errors, inadequate training, interpretation of weak results, understanding and resolving of indeterminate results, adherence to testing procedures and workload. These studies in addition to the strengthening of existing proficiency testing and quality assurance systems to regulate and monitor performance of HIV testing are needed as even a small error rate can result in a high number of misdiagnosed cases in context of high testing volumes.

This is of particular importance as Swaziland and other sub-Saharan governments have embraced the new UNAIDS (90–90–90) targets [20]. To achieve the first 90, that 90% of people infected with HIV should know their status, governments will require not only increased testing but also innovative and smarter testing strategies. In addition to embracing the 90–90–90 targets, treatment for all is now a public health standard in most countries, including Swaziland which has adopted the 2015 WHO guidelines in October 2016.

## Conclusions

The current findings showed an overall proportion of 0.6% false positives. With adoption of EAAA guidelines, ART initiation regardless of immunological criteria, by national governments including Swaziland in October 2016, there is a need to strengthen national HIV testing processes including proficiency testing. In addition to improving HIV testing quality, adoption of retesting prior to ART initiation would also allow identification of clients misdiagnosed previously to further reduce potential of initiating misdiagnosed clients on ART.

## Abbreviations

ART, antiretroviral therapy; EAAA, Early Access to ART for All; MaxART, Maximizing antiretroviral therapy for better health and zero new infection; MoH, Ministry of Health; NRL, National Reference Laboratory; RDT, rapid diagnostic test; WHO, World Health Organization.
